# Factors shaping local people’s perception of ecosystem services in the Atacora Chain of Mountains, a biodiversity hotspot in northern Benin

**DOI:** 10.1186/s13002-019-0317-0

**Published:** 2019-08-14

**Authors:** Fidèle Tchossi Moutouama, Samadori Sorotori Honoré Biaou, Boateng Kyereh, Winston Adam Asante, Armand K. Natta

**Affiliations:** 1 Kwame Nkrumah University of Science and Technology, University Post Office, Kumasi, Ghana; 2 Laboratory of Ecology, Botany and plant Biology (LEB), University of Parakou, 03 BP 125, Parakou, Benin; 3 Department of Natural Resource Management, Faculty of Agronomy, University of Parakou, Parakou, Benin

**Keywords:** Mountains, Ecosystem services, Perception, Atacora Chain of Mountains

## Abstract

**Background:**

Atacora Chain of Mountains (ACM) is the Benin part of a range of mountains lying from Benin to Ghana through Togo. It provides goods and services to people and is dominated by rural communities with heavy reliance on natural resources. The ACM may be threatened by the increasing resource needs and the possible shift in people’s livelihood priorities brought about by challenges and opportunities ushered in by modernization. This study sought to understand local people’s perception of the services derived from the ACM and the socio-demographic factors (age, gender, and level of urbanization of the place people live in) accounting for these perceptions.

**Methods:**

Face to face interviews with 12 focus groups in 3 municipalities were carried out to obtain a list of ecosystem services cited by the participants. At the individual level, 144 people (men, women, young, adult, and old people from either rural or urbanized areas) equally distributed in the 3 municipalities were asked whether they acknowledge each of the services cited by the focus groups. Logistic regressions were then used with generalized linear models (GLM) function in R to analyze the relationships between the probability of acknowledgement of a service by the respondents and their socio-demographic characteristics.

**Results:**

Local people perceived the ACM as a provider of provisioning, supporting, and cultural services but cited more provisioning services than the others. The factors considered in this study (gender, location, and age) were all predictors of ecosystem services perception in the ACM. Location influenced people’s perception of provisioning and supporting services such that respondents from rural municipalities were more likely to perceive provisioning and supporting services. This is because people in rural areas have a heavier reliance on natural resources. Gender was associated with respondents’ perception of supporting, provisioning, and cultural services. Women were more likely to perceive provisioning services and less likely supporting and cultural services. People in each category of gender have a perception of ES linked to their livelihood activities. Young people were less likely to perceive supporting services than adults and old people due to their less involvement in farming activities.

**Conclusions:**

This study showed that gender, location, and age predict local people’s perception of ecosystem services in the ACM and livelihood orientation is determinant. The omission of regulation and many of the supporting services questions the future of this mountain chain if its exploitation continues without any awareness and conservation measures.

## Background

Mountains carry complex and fragile ecosystems, highly differentiated climatic conditions, and vertical processes [1]. Because of the compression of climatic life zones with altitude and small-scale habitat diversity caused by different topoclimates, mountain regions are commonly more diverse than lowlands and are thus of prime conservation value [2]. For example, mountains host half of all 34 global biodiversity hotspots [3] and also harbor a high number of endemic species [4, 5]. Human well-being and progress toward sustainable development are vitally dependent upon the earth’s ecosystems [6]. Twenty percent of the world’s human population lives in mountains or at their edges [7]. During the era of very limited human population, people would harvest natural goods and enjoy services resulting from the simple functioning of the ecosystems they lived in. The growing population and the continuously modernizing world have led to land use management dictated by the growing per capita and total population needs. From the consumptive use of resources, many ecosystems have gone through the productive exploitation pattern to the extent of regime shifts. The use of land to produce goods and services represents the most substantial human alteration of the earth system [8]. The growing demands of services from mountain ecosystems by local people [9] has in some cases led to their unsustainable use over the long term [10]. Land use pressure puts mountain ecosystem integrity at risk in many parts of the world with industrial use, forest destruction, overgrazing, and inappropriate cropping practices leading to irreversible losses of soil and ecosystem function with increased environmental risks in both mountains and adjacent lowland [7]. Africa mountain ecosystems are known to provide goods and services to local people and therefore constitute the basis of their socio-economic and cultural development [11, 12]. Sub-Sahara Africa’s experience shows that significant population growth in mountain areas, together with unsuitable traditional farming and management practice, put natural resources under intense pressure [10]. The degradation of the earth ecosystems has drawn the attention of the scientific and international communities and resulted in studies such as the Millennium Ecosystem Assessment (MEA). The objective of the MEA was to assess the consequences of ecosystem change for human well-being and the scientific basis for action needed to enhance the conservation and sustainable use of those systems and their contribution to human well-being [2]. Since then, there has been a growing interest in the academic realm in ecosystem services (ES) but the emphasis has mainly been on the biophysical and economic aspects. Nonetheless, social studies can provide new insights into the valuation of ES, as well as into public opinion and the political climate with regard to the degradation or management of ecosystems [13]. Analysis of the socio-cultural preferences of different societal groups fosters awareness of the frequently neglected trade-offs in demands for ES and consequently provides information about the social dynamics surrounding ES [14]. Yet, while multiple disciplinary approaches should be integrated into ES assessments, non-economic social analyses have been lacking, leading to a knowledge gap regarding stakeholder’s perceptions of ES [15]. Policies impacting mountain ecosystems are sometimes made without reflecting the interests of local population which in the end affect the sustainability of conservation measures designed to benefit all stakeholders. Even though studies showed that multiple factors shape local people perception of ES [16–19], perception is context-specific and needs to be addressed as such. Besides, the way social, economic, and environmental changes drive perceptions may differ, from place to place making studies across space necessary for a fuller account of people’s perception of ES. Understanding people perception is important in designing effective environmental information and education campaigns.

**Fig. 1:**
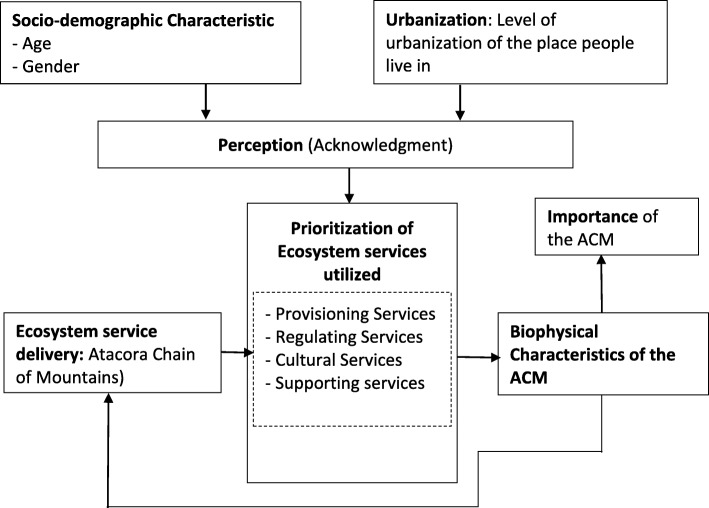
Conceptual framework on the link between socio-demographic factors, ecosystem services perception and the ACM. This study hypothesized that people’s gender, age, and the level of urbanization of the place they live in are associated to their awareness of each category of ecosystem service. The awareness was measured by whether a person acknowledges or not that the ACM provide a particular service. How people use and prioritize the different ecosystem services provided by the ACM is determined by their perception of these services. The perception of the ecosystem services and the use of these services can affect the biophysical characteristic of the ACM and therefore its importance and its long-term ability to provide services. For instance, if people are not aware of the role of mountain trees in carbon stock and the local climate, they will tend to cut trees for various uses without given the required attention to the long-term effect of deforestation on the climate and carbon stocks

Atacora Chain of Mountains is a portion of a range of mountains which lies from Benin to Ghana passing through Togo. The range of mountains is a refuge for moist and dry forest species and endemics and rare species [20–23]. Different ethnic groups have settled along the range of mountains over the time. These people have developed an interaction with the mountains and have influenced the ecosystems. They derive a wide range of ES for subsistence and commercial use. The population around the ACM is characterized by a fast growth rate (3.06%) with 79% of the people engaged in the primary sector [24] characterized by agriculture and natural resource exploitation. The range of mountains is exploited without any monitoring or regulation, and previous natural vegetation areas are cleared up for farming, housing, and commercial activities. Yet, harsh climates, uneven topography, and diversified geological and hydrological conditions make mountain ecosystems particularly vulnerable to inappropriate natural resource management practices and environmental degradation processes [1]. The ACM may be threatened by the increasing resource needs (driven by population growth), and the possible shift in people’s livelihood priorities brought about by challenges and opportunities ushered in by urbanization and modernization. There is therefore a need for an intervention that will ensure sustainable management and protection of the mountain’s ecosystem. Due to the fact that ecosystem goods and services are inherently public [25] coupled with the uncertainties created by the abovementioned threats, the effectiveness of any conservation action is linked to the level of inclusion of local people. To better take into consideration the spatial shifts in people’s priorities, local perception of the services derived from the ecosystem is of key importance. Theories in ethnobotany [26] demonstrate that socio-cultural and demographic traits such as gender, age, and literacy/formal educational level are all correlated with an individual’s level of plant knowledge and that urbanization decreases traditional and/or local ecological knowledge. The objective of this study was to assess the relationships between gender, age, urbanization, and ecosystem service perception through the following hypotheses: (i) women are more likely to perceive provisioning services than men, (ii) men are more likely to perceive supporting services than women, (iii) rural people are more likely to perceive provisioning services than people living in more urbanized areas, (iv) young people are more likely to perceive supporting and regulating services than old people, (v) old people are more likely to perceive cultural services than young people, and (vi) rural women are more likely to perceive provisioning services than women living in more urbanized areas. The assumed relationships between socio-demographic factors, ecosystem service perception, and the ACM are summarized by a conceptual framework (Fig. 1) as follows.

Perception can simply be defined as the way people see things, but The SAGE Encyclopedia of Qualitative Research Methods [27] provides a more comprehensive meaning of the concept: “Perception is a mode of apprehending reality and experience through the senses, thus enabling discernment of figure, form, language, behavior, and action. Individual perception influences opinion, judgment, understanding of a situation or person, meaning of an experience, and how one responds to a situation…. Perceptions are interpretations, and for most individuals, interpretations become their truth. Thus, perceptions are extremely powerful and influential in human thought and behavior…. Individuals and groups often “see” entities quite differently based on different life contexts and contingencies.” As such, perception is hereby referred to as the “spontaneous knowledge or awareness” of ES.

## Methods

### Study area

**Fig. 2:**
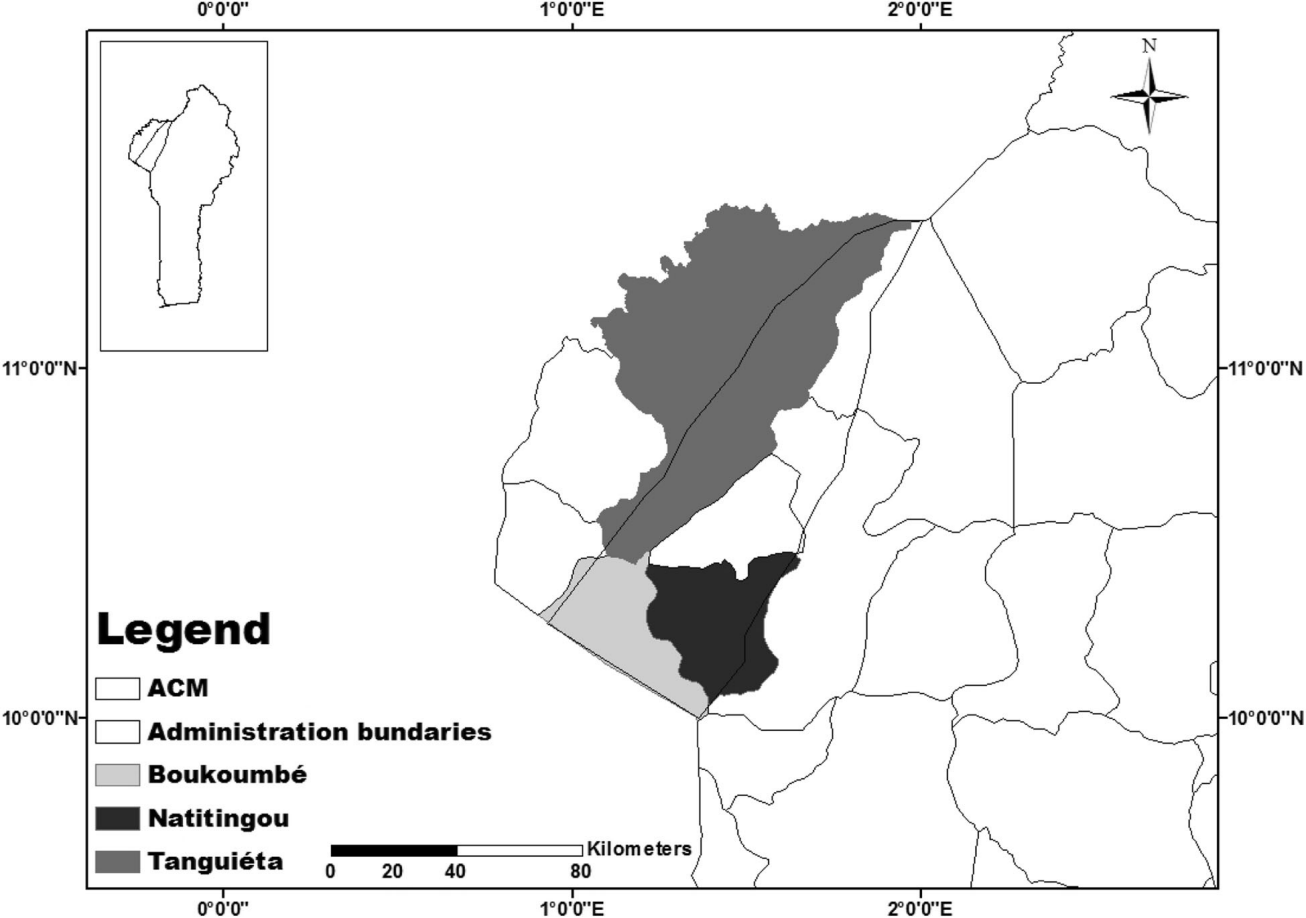
The Atacora Chain of Mountains (ACM) and location of the study municipalities

Our study area is the surroundings of the Benin part of a long chain of mountains, oriented NNE-SSW, which lies from Benin to Ghana passing through Togo. In Benin, the chain is in north-west in the Atacora department (Fig. 2). With an altitude ranging from 300 to 650 m [23], the ACM is located between 1° 00′ and 2° 00′ East and 10° 40′ and 11° 28′ North. The area has a tropical climate of a Sudanian type with one dry season (November to April) and one rainy season (April to October). Due to the presence of Atacora Chain, the annual rainfall which ranges between 1200 and 1350 mm is higher than the average of this climate type. The dry season is characterized by Harmattan, the north-easterly dry and hot wind blowing from the Sahara. The average annual temperature is 28 °C, and the relative humidity ranges from 27 to 83%.

### Site selection

The ACM crosses six municipalities, Natitingou, Toucoutouna, Tanguieta, Kerou, Kouande, and Boukoumbe. However, the investigation was carried out in three of the municipalities based on a stratified random sampling designed to capture a gradient of urbanization. Natitingou, the most urbanized of the three, is the capital of the department and is characterized by a well-patronized quarrying industry. Boukoumbe is the most rural and poorly accessible municipality where specialized mountain’s farming systems have been developed. Tanguieta offers an intermediary situation, being less urbanized than Natitingou but more advanced than Boukoumbe and where the proximity of the Pendjari National Park may offer different livelihood opportunities to rural people.

### Data collection

Local people perception of the benefits received from the mountains was assessed through face to face interviews. The interviews were conducted with four focus groups in each of the municipalities with the objective to get a list of ES that would then be used for the interviews with individuals. Within each municipality, the focus groups took place in four different localities. Two main questions were asked of the participants following Casado-arzuaga [9]: (i) do you think there is anything beneficial you derive from the mountains? and when the answer was yes, (ii) can you please cite them? Prior to these main questions, a short presentation on the concept of ES was made in the local language without providing detailed examples on ES related to the mountains. We avoided giving examples and asking closed questions in constituting the list in order to make sure we get the spontaneous citations of the services which, in our view, gives the true awareness of the services. Interviews were not recorded to encourage more open and honest answers [18]. Rather, there were two interviewers, one note taker and one member leading the discussions. The benefits were cited in local languages and sometimes required explanations from the participants to allow proper matching with the academic ES. The final list of services was the compilation of all the services cited in the 12 focus groups. This list was used for the questionnaire administrated to 48 people in each of the municipalities, leading to a total of 144 including men and women. The interviewees aged between 17 and 60 years old residing around the Atacora Chain of Mountain (Table 1). The questionnaires were structured in the following sections: the socio-demographic characteristics of the respondents and the perceptions of the importance of the mountains. The respondents were asked whether they think they receive anything beneficial from the mountains. When the answer to this question was yes, the respondents would be asked if they acknowledge each service in the list. The spontaneous acknowledgement (perception) or otherwise was then used as a binary variable for statistical analyses. To make sure the concept of ES was well understood, each benefits mentioned on the list gotten from the focus groups was explained in the local languages to the respondent before the task of acknowledgement was performed [28].

**Table 1:** Sample composition according to municipality, gender, and age

		Municipality			
		Tanguieta	Natitingou	Boukoumbe	Total
Gender	Women	17	8	5	30
	Men	31	40	43	114
Age	Age < 40	18	23	12	53
	40 ≤ age ≤ 60	28	21	34	83
	Age > 60	2	4	2	8
Origin	Native	47	37	48	132
	Non-native	1	11	0	12
Total		48	48	48	144

The MEA [29] provided a widely adopted definition of ES where ES is simply defined as the benefits people obtain from ecosystems. The MEA delineated four categories of ES (Table 2): provisioning services (the products obtained from ecosystems), regulating services (the benefits obtained from the regulation of ecosystem processes), cultural services (the nonmaterial benefits people obtain from ecosystems through spiritual enrichment, cognitive development, reflection, recreation, and esthetic experiences), and supporting services (those that are necessary for the production of all other ecosystem services). The Economics of Ecosystems and Biodiversity (TEEB) also proposes four categories of ES, namely provisioning, regulating, habitat, and cultural and amenity services [30]. TEEB considers habitat rather than the supporting services of the MEA as a category given that supporting services are a subset of ecological processes. Haines-young et al. [31] define ES as the contributions that ecosystems make to human well-being and refers specifically to the ‘final’ outputs or products from ecological systems. As such, the Common International Classification of ES (CICES) focuses on provisioning services, regulating and maintenance services and cultural services to avoid double counting in the valuation which may result from including supporting services. The revised report [32] distinguished services from goods and benefits (suggested to be named products) and stated that ecosystem goods and benefits are things that people create or derive from the final ES.

**Table 2:** Ecosystem services categories according to MEA

Provisioning services	Regulating services	Cultural services	Supporting services
▪ Food	▪ Climate regulation	▪ Spiritual and religious	▪ Soil formation
▪ Fresh water	▪ Disease regulation	▪ Recreation and ecotourism	▪ Nutrient cycling
▪ Fuelwood	▪ Water regulation	▪ Aesthetic	▪ Primary production
▪ Fiber	▪ Water purification	▪ Inspirational	
▪ Biochemical	▪ Pollination	▪ Educational	
		▪ Sense of place	
		▪ Cultural heritage	

Notwithstanding the fact that supporting services are increasingly excluded from the categories of services and rather considered as functions of the ecosystem [30, 31], we chose to use the four categories as proposed by the MEA in order to see how knowledgeable people are with regard to this important group that sustains the others. We also considered ecosystem goods and services as ecosystem services.

### Data analysis

Data were grouped according to location (municipality), gender, and age on the one hand and according to ecosystem categories on the other hand. The age variable was categorized into three groups: young, adults, and old [33, 34]. Simple logistic regression was used to test whether the probability that a respondent acknowledges a service (provisioning, regulating, supporting, or cultural) the ACM provides them is affected by their gender, age, and location (municipality) (independent variables). We used a GLM function with a binomial distribution for each of the dependent variables [35]. For significant predictors, the risk factor was used to determine the direction of the prediction. When the risk factor is greater than 1, there is a positive relationship and the respondent is more likely to perceive the service.

## Results

### Differences in the number of perceived benefits captured under the various ecosystem service categories

In each of the communities surveyed, all respondents stated that they derive some form of ecosystem service from the ACM and the perceived services included three out of the four categories of ES: provisioning, supporting, and cultural. None of the respondents mentioned a regulating service. The most cited category was provisioning services whose identified services included herbage for grazing, quarry materials, fuelwood, game, food, and medicinal plants. It was followed by cultural services where two benefits were stated and finally supporting services with one benefit, soil quality. The two cultural services cited by interviewees were tourism and spiritual benefits. Table 3 provides the local expressions of ES and the provided translation.

**Table 3:** Expressions of ecosystem services by focus groups and equivalent MEA services

Explanation by the participants: most heard sentences related to cited services in focus groups	Translated expressions for the service by the research team	MEA category
• “We collect small stones from the mountains that we sell to people who construct houses in town” • “We extract stones from the mountains that we polish and sell to people who want to decorate their houses here and mostly merchants who go to the capital” • We break middle size stones from the mountains into small pieces and we sell them for construction”	Quarry materials	Provisioning services
• “Women collect wood from the mountains to cook and sell” • “When we clear land for cropping, we transformed the woody plants species into charcoal”	Fuelwood	
• “There are animals on the mountains and we use traps to catch them for consumption and for sale”	Game	
• “There are different fruit-trees on the mountains that we harvest for consumption and sale” • “We harvest wild yam from the mountains that we eat during the period of food shortage” • “On the mountains, there is often wild honey that we harvest for consumption and sale” • “There are so many foodstuffs we get from the mountains”	Food	
• “There are numerous plant species that we use to treat many diseases that are only found on the mountains nowadays”	Medicinal plants	
• “Right now, soils on the valley have low crop yield. If you do not have money to buy fertilizers, there are crops that you can produce on the mountains that will have higher yield than in the valleys” • “Because of the current delay in rainfall, there are crops like sorghum that do not thrive on the mountains because mountain soil is often more humid”	Soil quality	Supporting services
“People from other places come here to visit our mountains”	Tourism	Cultural services
“We perform our family ceremonies on the mountains” “We have our tribe fetishes on the mountains”	Spiritual use	

### Factors predicting the perception of ES along the ACM

The results of logistic regression models (see Table 4) showed that all the factors considered in this study, gender, age, and location (municipality), are predictors of ecosystem services’ perception. The perception of provisioning services was significantly predicted by respondents’ gender and location while the three factors significantly predict the perception of supporting services and only gender predicts the perception of cultural services. Also, all factor interactions did not significantly predict the probability of perception of any category of service.

**Table 4:** Logistic regression of gender, age and location factors on the acknowledgment of ecosystem services. This table provides the results of the logistic regression showing the factors that significantly predict people’s perception of ES and the direction of the relationship

	Estimate	Std. error	*z* value	Pr(> |*z*|)	Risk factor	Estimate	Std. error	*z* value	Pr(> |*z*|)	Risk factor	Estimate	Std. error	*z* value	Pr(> |*z*|)	Risk factor
	Provisioning services					Supporting services					Cultural services				
(Intercept)	2.2851	0.5364	4.26	2.05e−05 ***	9.83	1.7539	0.4237	4.1390	3.49e−05 ***	5.78	− 0.02404	0.31579	− 0.076	0.93931	0.98
Ageold	− 1.0074	1.0661	− 0.945	0.3447	0.37	1.8483	1.6061	1.1510	0.2498	6.35	2.58853	1.32112	1.959	0.05007 .	13.31
Ageyoung	0.2335	0.5183	0.45	0.6523	1.26	− 1.7779	0.4671	− 3.8060	0.000141 ***	0.17	− 0.02061	0.41893	− 0.049	0.96076	0.98
GendWoman	2.2294	1.0601	2.103	0.0355 *	9.29	− 4.6256	1.1660	− 3.9670	7.28e−05 ***	0.01	− 2.50069	0.81879	− 3.054	0.00226 **	0.08
DistNatitingou	− 1.5355	0.6449	− 2.381	0.0173 *	0.22	− 1.1479	0.5374	− 2.1360	0.032664 *	0.32	− 0.57621	0.4672	− 1.233	0.21746	0.56
DistTanguieta	− 1.1908	0.6699	− 1.777	0.0755 .	0.3	− 0.0022	0.5911	− 0.0040	0.9970	1	− 0.33433	0.46407	− 0.72	0.47126	0.72

The intercept is the predicted value of the response when all predictors are 0; the estimate are the slopes; Std. error is the standard error. Signif. codes: 0 “***”, 0.001 ‘**’ 0.01 ‘*’ 0.05 ‘.’ 0.1 ‘ ’ 1

The probability of respondents to acknowledge a listed provisioning service decreases with Natitingou (*p* = 0.0173) for the municipality (location) factor while it increases with women (*p* = 0.0355) for the gender factor. With a risk factor of 9.29, women are more likely to acknowledge provisioning service. Adding one woman would increase the number of acknowledgements of provisioning services by 9.29. In contrast to Boukoumbe, respondents from Natitingou and Tanguieta municipalities are less likely to perceive provisioning services (risk factor < 1). The probability of perception of supporting services decreases with Natitingou municipality (*p* = 0.0326), women (*p* = 07.28 × 10^-5^), and young people (*p* = 0.0001). Respondents from Tanguieta and Boukoumbe for municipality factor are more likely to perceive supporting service while women for gender factor and young people for age factor are less likely to perceive these services. The risk factor of old respondents is 6.35. The relationship between gender and cultural services is such that the probability of respondent to perceive a service of this category decreases as the number of women increases (*p* = 0.0023). Women are less likely to perceive cultural services (risk factor < 1).

The percentage of respondents acknowledging the ecosystem services derived from the ACM under significant factors (see Table 5) showed that a higher percentage of respondents in Boukoumbe perceived provisioning services than in Natitingou, while Tanguieta fell in between. Women did not perceive some provisioning services (grass/herbage and medicinal plant), but for services like fuelwood and food, they had higher frequency. Similar to the trend in provisioning services, Boukoumbe had a higher percentage of perception of supporting ES followed by Tanguieta and Natitingou. For this category, men had a higher percentage than women and young people lower percentage than adult and old. For cultural services, men had a higher percentage of perception than women.

**Table 5:** Percentage of respondents citing the ecosystem services derived from the ACM. This table provides the percentage of respondents citing services specific per significant factors

Percentage of respondents citing the services									
	Provisioning services						Supporting services	Cultural services	
Factors	Grass/herbage	Quarry materials	Fuelwood	Game	Food	Medicinal plants	Soil quality	Tourism	Spiritual use
Municipality									
Tanguieta	–	29.2	62.5	–	56.3	52.1	47.9	10.4	18.8
Natitingou	20.8	43.8	50	2.1	43.8	47.9	39.6	4.2	29.2
Boukoumbe	35.4	–	62.5	18.8	58.3	62.5	70.8	–	45.4
Gender									
Men	23.68	23.68	56.14	8.77	43.86	54.39	64.91	0.06	36.84
Women	–	20	63.33	–	53.33	40	3.33	–	6.67
Age									
Young (< 40)	9.43	28.3	32.08	5.66	30.19	30.19	18.87	9.43	11.32
Adult (40–60)	25.3	19.28	49.4	7.23	56.63	55.42	68.67	2.41	34.94
Old (> 60)	37.5	–	–	12.5	–	–	50	–	37.5

## Discussion

The current study investigated local people’s perception of ES and the socio-demographic factors associated with these perceptions along the ACM. All the respondents perceived the ACM as a provider of ES. Even though the interviewees had no prior exposure to the concept of ES, they could state at least one benefit under one category of ES that they derive from the ACM. Previous studies also showed that people are often aware of ES even when they do not use the scientific concept of “ecosystem service,” Examples of such studies are from Southeast Asia [16, 36], Spain [9], and other parts of Europe [37]. It was also observed that there were differences in the level of local people’s perception of the various ES categories. People along the ACM identified more provisioning services and cultural services than supporting and regulating services. Similar results were reported by Lamarque et al. [37] who showed that more visible services were spontaneously identified. McNally et al. [19] and Hartel et al. [18] also found that people placed a significantly higher level of importance on provisioning ES than the other services. In contrast to our work, Martin-López et al. [36] and López-Santiago et al. [14] found that regulating services are reported more often than provisioning services, even though the latter are easier to physically identify. According to the authors, this is due to the high level of awareness of air pollution in Spanish cities where the studies took place. Another important finding of the study is that municipality, gender, and age shaped local people’s perception of supporting services, municipality and gender shaped local people’s perception of provisioning service, and only gender affects the perception of cultural services. The fact that respondents from Natitingou were less likely to perceive provisioning and supporting services while those from Boukoumbe were more likely to perceive these services may result from the level of urbanization of the municipalities. Being the capital of the department and most urbanized, Natitingou and its surrounding villages have a more modernized lifestyle. They rely less on the natural ecosystem for food, firewood, and medicinal plants. As the area is getting urbanized, the use of gas for cooking and heating may be increasing and there is probably a shift in eating habits. Also, people have easier access to hospital and can afford modern health care services, which will then reduce their reliance on traditional medicine and thus medicinal plants. The highest citation with regard to quarry materials in Natitingou may be explained by the fact that people in this city are noted for quarrying and sale of stones for construction and decoration all over Benin. Boukoumbe is the most rural of the three municipalities with therefore a higher reliance to natural resources. This leads to a higher likeliness to perceive provisioning services such as fuelwood, food, and medicinal plants. With regard to supporting services, respondents from Boukoumbe are more likely to perceive them because they have less available land for agriculture (the municipality is confined within hills and mountains). The place is the most rural of the three municipalities, and people depend on mountains agriculture for their subsistence. The results suggest that the level of urbanization may play a role in people’s perception of provisioning services as found by previous studies [14, 15, 36, 38].

The gender factor affects the perception of provisioning, supporting, and cultural services. Considering provisioning services, a higher percentage of women cited food and fuelwood while men cited game, grass for grazing, and medicinal plants more than the other provisioning services. In the Atacora department, rural women are in charge of collecting firewood from the forest for domestic use, and in the process, they also harvest wild fruits for consumption. Moreover, as wood in the lowlands get depleted, women progressively reach formerly inaccessible places in the mountains searching for firewood and this may deepen their appreciation of the role of the ACM in providing these two services. Hunting and grazing are male-dominated activities. Participants in each category of gender factor can therefore be said to have a perception of ES linked to their livelihood activities. Likewise, along the ACM, men own land and take the major management decisions in farming [39]. This may explain why they are more likely to perceive the supporting services (of better soil quality). Traditional ceremonies are also often entrusted to men who therefore perceive the ACM as a place for spiritual activities, causing gender to shape the perception of cultural services. These observations support the view that people tend to assign the greatest priorities to services most closely linked to their livelihood [19] and that their perception could also be determined by the prevailing conditions or level of awareness of such conditions around their place of residence [14]. The age factor also affects the perception of supporting services. Along the ACM, young respondents are less likely to perceive supporting services (better soil quality on the ACM) than adults and old people. This may be explained by the fact that young people are probably less involved and experienced in farming. The youth’s better exposure to science may account for the difference in perception. The influence of gender and age on the perception of ecosystem services was also found by previous studies, e.g., [36, 38]. Significant services scientifically known to be provided by the presence of the mountain chain were not cited by participants of focus groups. For example, the ACM causes higher rainfall conditions (climate regulation), hosts a diversity of land uses and plant and animal species [23], and serves as source of many rivers [40] Moreover, Atacora chain is located in the Sudanian Center of regional endemism [41]. It is significant for its phytomass and subsequently carbon sink and storage. The fact that many of the supporting and regulating services were not mentioned corroborates the idea that people first mentioned what they directly feel or receive from the ecosystems. Because the supporting services are basically processes, they sustain the delivery of the other services that are more obvious to people. The uncertainty this raises is what is likely to happen to the ecosystem if it is exploited and managed only on the basis of more tangible services.

## Conclusions

This study may be the first to investigate the perception of the local communities on ES of the ACM, a hotspot of biodiversity in Benin. Local people identified a range of benefits pertaining to three out of the four categories of ES known to science. However, they were heavily skewed towards provisioning services to the extent that only soil quality was cited for supporting service and no regulating service was cited. People therefore perceived tangible services more than intangible services raising the concern that if nothing is done to raise the awareness of local people on the intangible services of the ACM, there is a risk that people may pay less attention to its conservation needs. Multiple factors (location, gender, and age) influenced people’s perceptions of ES, and these factors tended to be associated with the respondent’s livelihoods. This paper showed that prior to any policy or conservation action, it may be necessary to assess and work on local people’s perception of the ecosystem services related to their livelihoods.

## Data Availability

The datasets used and analyzed during the current study are available from the corresponding author on reasonable request.

## Abbreviations

ACM: Atacora Chain of Mountains
CICES: Common International Classification of Ecosystem Services
ES: Ecosystem services
FAO: Food and Agriculture Organization
GLM: Generalized linear model
INSAE: Institut National de la Statistique et l’Analyse Economique
MEA: Millennium Ecosystem Assessment
TEEB: The Economics of Ecosystems and Biodiversity
